# Integrating sociodemographic index and Bayesian modeling in liver cancer research: opportunities and limitations

**DOI:** 10.1097/JS9.0000000000002997

**Published:** 2025-07-08

**Authors:** Lingling Meng, Yupeng Di

**Affiliations:** a Department of Radiation Oncology, Senior Department of Oncology, The First Medical Center of PLA General Hospital, Beijing, China; b Department of Radiotherapy, Air Force Medical Center, PLA, Beijing, China

*Dear Editor*,

We read with great interest the comprehensive study by Wang[1], which analyzed the global burden of primary liver cancer (PLC) in populations aged 55 years and over. This work fills a critical gap by shedding light on etiology-specific trends, demographic disparities, and future projections, providing invaluable guidance for targeted public health strategies in an aging global population. The integration of sociodemographic index (SDI) stratification and Bayesian age-period-cohort (BAPC) modeling provides policymakers with robust evidence, particularly with regard to the rising threat posed by metabolic drivers (NASH/alcohol) and the projected U-shaped incidence curve up to 2050.

However, several methodological limitations must be addressed to improve future analyses. In addition, to ensure transparency and integrity in the reporting of scientific communication, this manuscript adheres to the TITAN 2025 guideline for the disclosure of artificial intelligence in biomedical research[2].

First, the 10-year age groupings (e.g., 55–59, 60–64, and 65–69 years) obscure important differences within the ≥75-year age group – the group with the highest and fastest-growing PLC burden. Combining all individuals aged 75 years and over into a single category fails to capture nuances in comorbidity profiles, treatment accessibility, or biological aging across subpopulations (e.g., 75–79 vs. ≥85 years), which could potentially mask vulnerabilities in the “oldest old.” Second, although NASH-related PLC increased globally, regional variations in important metabolic drivers (e.g., diabetes prevalence and obesity rates) were not considered. This omission could lead to an overestimation of NASH’s independent contribution, as the combined effects of these factors and viral hepatitis could make it difficult to attribute the cause in regions with a high SDI. Third, the BAPC model assumes static future population structures, but this overlooks the potential for disruption from migration, conflict, or pandemics (e.g., war-induced refugee flows in Europe or fertility decline in East Asia). Such dynamic shifts could significantly alter age distributions by 2050, thus undermining the validity of predictions.

Despite these limitations, this study is pivotal in mapping the evolving PLC landscape. Its focus on metabolic etiologies and aging-driven burden highlights the urgent need for region-specific interventions, ranging from viral hepatitis control in low-SDI settings to lifestyle modifications in high-SDI nations. We commend the authors for this timely work and encourage future studies to incorporate finer age stratification, comorbidity adjustments, and dynamic demographic modeling.

## Data Availability

The data supporting the findings of this study are available upon request.
